# Change blindness in pigeons (*Columba livia*): the effects of change salience and timing

**DOI:** 10.3389/fpsyg.2015.01109

**Published:** 2015-08-03

**Authors:** Walter T. Herbranson

**Affiliations:** Department of Psychology, Whitman College, Walla WallaWA, USA

**Keywords:** change detection, change blindness, attention, pigeon, timing

## Abstract

Change blindness is a well-established phenomenon in humans, in which plainly visible changes in the environment go unnoticed. Recently a parallel change blindness phenomenon has been demonstrated in pigeons. The reported experiment follows up on this finding by investigating whether change salience affects change blindness in pigeons the same way it affects change blindness in humans. Birds viewed alternating displays of randomly generated lines back-projected onto three response keys, with one or more line features on a single key differing between consecutive displays. Change salience was manipulated by varying the number of line features that changed on the critical response key. Results indicated that change blindness is reduced if a change is made more salient, and this matches previous human results. Furthermore, accuracy patterns indicate that pigeons’ effective search area expanded over the course of a trial to encompass a larger portion of the stimulus environment. Thus, the data indicate two important aspects of temporal cognition. First, the timing of a change has a profound influence on whether or not that change will be perceived. Second, pigeons appear to engage in a serial search for changes, in which additional time is required to search additional locations.

## Introduction

One fundamental consequence of the temporal aspects of cognition is the notion of change. Change differs from the related concepts of difference and motion in part because of the central role played by time (see Rensink, 2002). The world may become an importantly different place as time passes, and the ability to detect such changes across time must be fundamental to survival: a changed environment may require different sorts of responses. On a behavioral level, it is well established that the appearance of a discriminative stimulus elicits different behavior from an animal well-trained in the relevant contingencies. The concept is also true on a basic physiological level. Phasic receptors are one concrete indicator of the fundamental importance of change detection. Many sensory receptors respond not to the presence or absence of a stimulus (as tonic receptors do), but to any change in the stimulus environment (Knibestol and Valbo, 1970). By responding specifically to changes, phasic receptors create a neural signal that enables individuals to notice and attend to novel aspects of the world as they appear. Thus, change detection is a fundamental process of temporal cognition that seems to be built into the nervous system at its most basic level. Yet paradoxically, changes (even important ones) are not always detected.

One somewhat surprising illustration is the phenomenon of change blindness. Change blindness occurs when a clearly visible change to a stimulus display goes unnoticed. One particularly striking example of change blindness has been provided by Simons and Levin (1998). An experimenter stopped unsuspecting individuals on a college campus to ask for directions. During the ensuing conversation, two confederates carrying a door walked between the two conversants, and during the brief visual interruption the experimenter was replaced by a different person. About half of their participants did not notice the change in their conversation partner. Surprisingly, change blindness can occur even when an individual is looking directly at the location of the change, and when a participant is expecting and actively searching for changes (see Simons and Ambinder, 2005).

Change blindness, of course, can occur under a variety of circumstances and with a diverse range of stimuli. A convenient way of studying change blindness in the laboratory is the “flicker task,” developed by Rensink et al. (1997). They presented participants with two continuously alternating images, with consecutive presentations separated by a brief, blank inter-stimulus interval (ISI). The images were identical with the exception of a single feature, and participants were instructed to search for the difference as they alternated. Participants had difficulty finding even large changes, and normally required many repetitions before eventual successful identification. In contrast, when the same images were presented without the ISI, the change was immediately apparent. Thus, the timing of a change has a powerful influence over whether or not it will be detected. The difference in change detection between trials with and without an ISI provides a convenient and specific operational definition of change blindness, and underscores the importance of timing in change detection.

One of the appealing features of the flicker task is that it can be implemented in a laboratory setting, and Herbranson et al. (2014) developed a variation of the task to investigate a possible change blindness effect in pigeons. They presented pigeons with stimulus displays consisting of randomly generated lines across three response keys. As in other versions of the flicker task, alternating displays were identical except for one feature (a single line that was present in one display but absent in the other), and pecks to the location of the change were reinforced at the end of a trial. Pigeons displayed the expected change blindness effect, in that accuracy was better on trials with no ISI than on trials with an ISI between consecutive displays. Their results also showed some other complex patterns reflecting the importance of time. In particular, the duration of the ISI had a powerful influence over the magnitude of the change blindness effect. As the ISI was shortened, accuracy on ISI trials rose toward the higher accuracy of no-ISI trials. In addition, pigeons showed evidence of using a serial search strategy over time. As the number of repetitions of the change increased, accuracy also increased, as did the effective search area. With few repetitions, pigeons produced overall low accuracy, and could reliably detect changes appearing on only two of the three response keys. With more repetitions, accuracy was higher overall, and better than chance on each of the three response keys.

Pigeons from Herbranson et al. (2014) showed accuracy that was above chance, but not always particularly high (especially on trials that featured an ISI). Nevertheless, some aspects of the procedure increased overall accuracy by systematically increasing accuracy on the more difficult ISI trials: number of repetitions, and ISI duration. It is likely that there are numerous other factors that would similarly influence change detection accuracy. Another plausible way to improve performance is to manipulate the salience of the displayed change. Smilek et al. (2000) used a flicker paradigm with alternating displays consisting of arrays of block characters. As is usually the case, one character differed between displays, and the change was characterized as either large or small, depending on the number of line features that differed. For example, a change of a character from F to L (three features) was considered a large change, whereas a change from F to E (one feature) was a small change. Their human participants were faster to detect changes involving more features than they were to detect changes involving fewer features.

In the modified pigeon version of the flicker task developed by Herbranson et al. (2014), the possible change locations are limited and fixed, corresponding to the three keys in an operant chamber. A change in any spatial location (i.e., on any particular key) is therefore likely to be roughly as salient as any other: they are the same size, brightness, color, and pecking on each has been reinforced with approximately equal frequency. However, the discrete stimulus features (lines) do permit one to make a change more prominent using the same logic as Smilek et al. (2000): by increasing the number of line features that constitute a change. Whereas Herbranson et al. (2014) presented two successive displays that differed by a single line feature on one key, the procedure is not limited to changes involving a single feature; up to eight changes (all of the possible line features) can be made to change on a single key. A difference of a single feature on a key would presumably be a smaller or more subtle change than a difference involving multiple features. As the number of changes increases, one would expect change detection to become proportionally easier, producing better accuracy, and requiring fewer repetitions.

## Materials and Methods

### Animals

Four White Carneaux Pigeons (*Columba livia*) were purchased from Double-T Farm (Glenwood, IA, USA). Each bird was fed mixed grain and maintained at 80–85% of free-feeding weight to approximate the condition of healthy wild birds (Poling et al., 1990). Birds were housed in individual cages in a colony room with a 14:10-h light: dark cycle and had free access to water and grit. All four had previous experiences with a serial response time task (Herbranson and Stanton, 2011) and a change detection task (Herbranson et al., 2014). Animal care and all procedures described below were approved by Whitman College’s Institutional Animal Care and Use Committee.

### Apparatus

Four identical BRS/LVE operant chambers were used. Each had three circular response keys (2.5 cm in diameter) located in a horizontal row on the center of the front wall and a food hopper located directly below the middle key. A houselight located on the front wall, directly above the middle key, was illuminated for the duration of each experimental session.

### Stimuli

Stimuli consisted of straight white lines back-projected onto each response key using stimulus projectors (Industrial Electronic Engineers, Van Nuys, CA, USA) that had been retrofitted with LED light sources (Martek Industries, Cherry Hill, NJ, USA). The LED light modifications were necessary because their onset and offset times (∼30 μs) are much faster than incandescent bulbs, allowing for precise control of even very fast stimulus presentations and ISIs. The three keys each could display up to eight radial lines, with each line spanning the full diameter of the key. The lines appeared at evenly spaced orientations corresponding to 0.0, 22.5, 45.0, 67.5, 90.0, 112.5, 135.0, and 157.5° from vertical. On each trial, a base stimulus was generated according to the following parameters: each of the eight lines on each of the three keys independently had a 0.5 chance of being present and a 0.5 chance of being absent. Consequently, each stimulus could consist of anywhere from 0 to 24 lines across the keys (0–8 per key). A modification of that base stimulus was then generated by reversing the display status of 1, 2, 4, or, 8 of the lines on a single key, depending on the experimental condition (see below). If the line to be reversed was present in the base display, then it was not present in the modified display. Conversely, if it was not present in the base display, then it was present in the modified display. The number of changes in the stimulus presentation was generated randomly, and each change was equally likely to occur in any of the eight orientations on the key.

Each trial consisted of alternating 250-ms presentations of the original and modified displays. The alternating displays were presented 1, 2, 4, 8, or 16 times (randomly determined on each trials with *p* = 0.2 for each). Each presentation of the original display was followed by the modified display and each presentation of the modified display was followed by either by a repetition of the original display or a trial-terminating display consisting of three white key lights (if and only if it was the final repetition of the trial).

**FIGURE 1:**
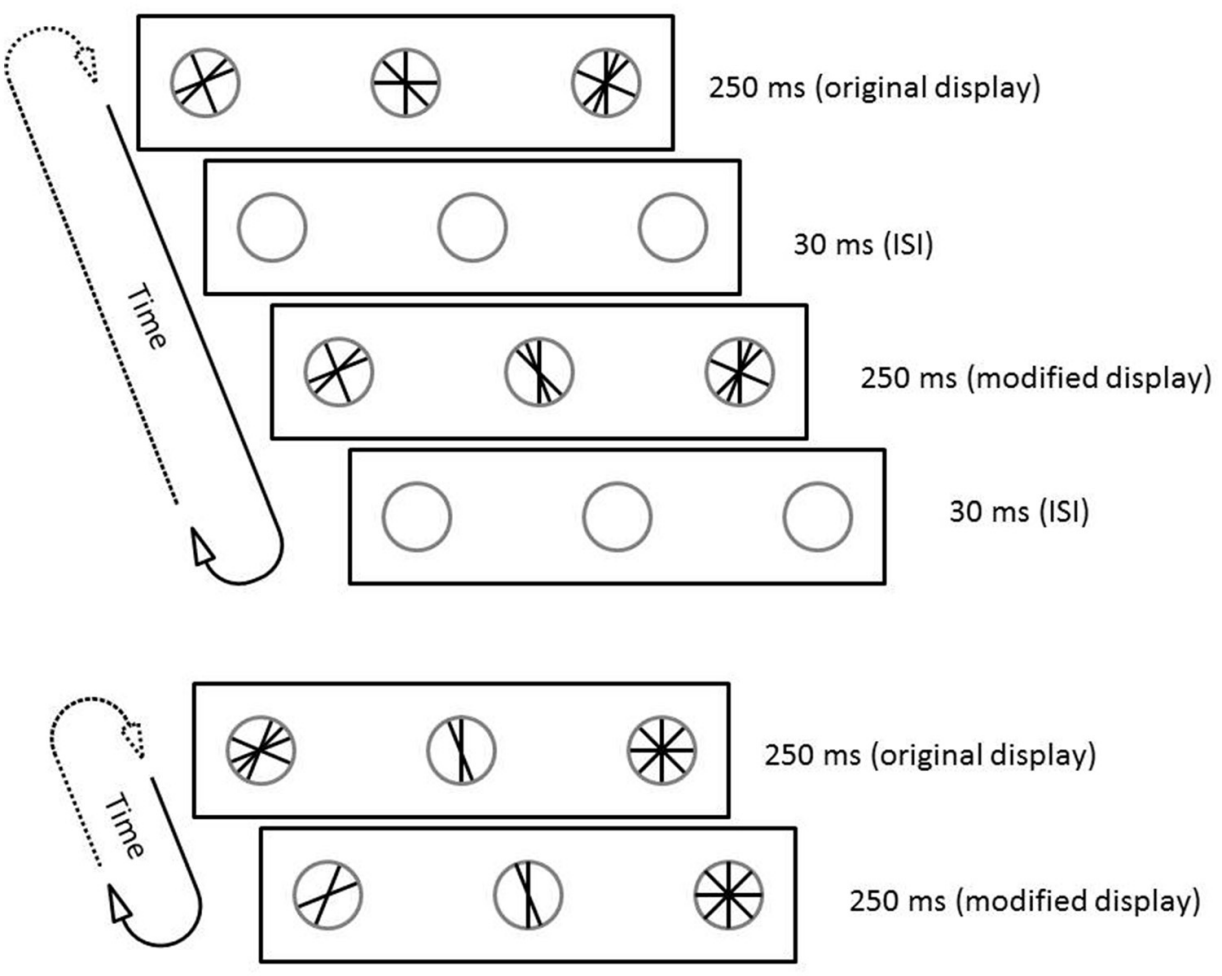
**Structure of a trial with an inter-stimulus interval (ISI; **top)** and without an ISI (bottom).** Both trials depict a change of two line features. **(Top)** the change is on the center key. **(Bottom)**, the change is on the left key.

Half of the trials presented the two alternating displays with no time delay in between. The modified display was presented immediately after the base display, so that there was no time when one of the two displays was not present on the response keys until the end of the trial. The other half of the trials contained a 30 ms ISI between the two displays, during which the keys were completely dark, and no lines were visible. The ISI was then followed immediately by the modified display. Thus, on trials with an ISI, the same number of repetitions took longer because each 250-ms stimulus presentation was followed by an ISI delay. The 30 ms ISI duration was selected based on the results from Herbranson et al. (2014) in order to produce an intermediate level of accuracy on ISI trials so that changes in accuracy could not be masked by floor or ceiling effects. **Figure 1** depicts two sample trials (both featuring two changes on the critical key), one with an ISI and one without.

### Procedure

The experiment was conducted daily over four blocks of 10 days each (40 days total). Each session consisted of 120 trials, each separated by a 5-s intertrial interval (ITI). During this ITI, the computer program generated original and modified displays, as well as determined the number of repetitions and whether to include an ISI. Pecks during stimulus presentation were not recorded and had no programmed consequences. Following completion of the entire stimulus display, all three keys were uniformly illuminated with white light, and the first peck on any key was automatically recorded. If the peck corresponded to the location of the stimulus change, then the bird was presented with approximately 3-s access to mixed grain (this varied between birds in order to maintain individual running weights). If the peck corresponded to either of the other two, unchanging locations, a 10-s error signal was presented, during which the houselight flashed on an off every 0.5-s. After either the reinforcement or the error signal, the session continued with a normal ITI, followed by the next trial.

### Conditions

The procedure during each block was identical with the exception of the number of changes displayed on the critical key during each trial. During the first block, a single line feature was reversed on each trial, regardless of any other stimulus characteristics (ISI, number of repetitions, etc.) This was the baseline condition, and paralleled the procedure from Herbranson et al. (2014), with the exception of the ISI duration. The subsequent three blocks displayed changes consisting of 2, 4, and finally 8 reversed line features on the critical key on each trial.

Because all four birds had previous experience on a slightly different version of the flicker task, no pretraining was necessary and data collection could begin immediately.

## Results

A 4 (changes: 1, 2, 4, 8) × 5 (repetitions: 1, 2, 4, 8, 16) × 2 (ISI: present, absent) × 10 (session: 1–10) repeated measures ANOVA was run on average change detection accuracy. The main effect of session was not significant, nor were any of the interactions involving session, *F* < 1.526, *p* > 0.061. These results indicate that performance was relatively stable across the 10 days that constituted each condition. The remaining analyses therefore consider the other factors collapsed across days.

**FIGURE 2:**
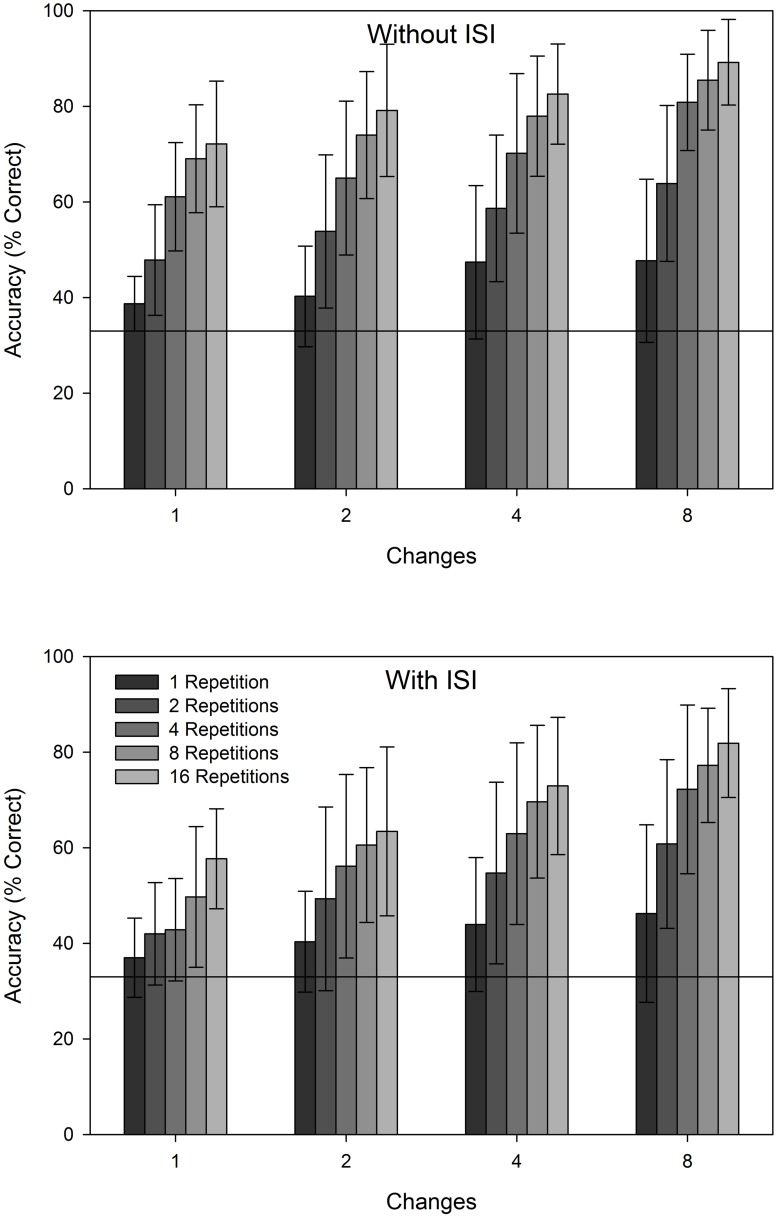
**Change detection accuracy over the 10 days of each condition as a function of number of changes, number of repetitions, and ISI presence.** Error bars depict 95% confidence intervals.

All three experimental factors (changes, repetitions, and ISI) yielded significant main effects, and the influence of each variable can be seen in **Figure 2**. The main effect of changes indicates that accuracy was better when there were more features that changed on a trial, *F*(3,15) = 41.953, *p* < 0.001, $\eta_{p}^{2}$ = 0.894. Mean accuracy increased as number of changes increased from 1 (*M* = 51.80, SE = 3.45) to 2 (*M* = 58.19, SE = 5.33) to 4 (*M* = 64.08, SE = 5.53) to 8 (*M* = 70.54, SE = 4.89). The main effect of repetition indicated that accuracy was better when more repetitions of the stimulus displays were presented, *F*(4,20) = 51.329, *p* < 0.001, $\eta_{p}^{2}$ = 0.911. Mean accuracy increased as number of repetitions increased from 1 (*M* = 42.68, SE = 4.45) to 2 (*M* = 53.87, SE = 5.83) to 4 (*M* = 63.90, SE = 5.40) to 8 (*M* = 70.44, SE = 4.78) to 16 (*M* = 74.87, SE = 4.48). Finally, the main effect of ISI indicated that accuracy was better when an ISI was absent (*M* = 65.23, SE = 4.27) than when it was present (*M* = 57.07, SE = 5.31), *F*(1,5) = 25.852, *p* = 0.004, $\eta_{p}^{2}$ = 0.838. This final main effect constitutes a basic replication of the change blindness effect seen in previous experiments using the flicker task.

In addition to the main effects reported above, all three 2-way interactions were significant: changes × repetition, *F*(12,0) = 2.287, *p* = 0.018, $\eta_{p}^{2}$ = 0.314; changes × ISI, *F*(3,15) = 5.677, *p* = 0.008, $\eta_{p}^{2}$ = 0.532; and repetition × ISI, *F*(4,20) = 7.806, *p* = 0.001, $\eta_{p}^{2}$ = 0.610. Note that the second of these 2-way interactions was particularly important for our purposes, as it indicates that the additional changes increased accuracy on ISI trials more than they did on no-ISI trials, thus decreasing the magnitude of the change blindness effect. Finally, the 3-way interaction was not significant, *F*(12,60) = 1.498, *p* = 0.150, $\eta_{p}^{2}$ = 0.230.

**FIGURE 3:**
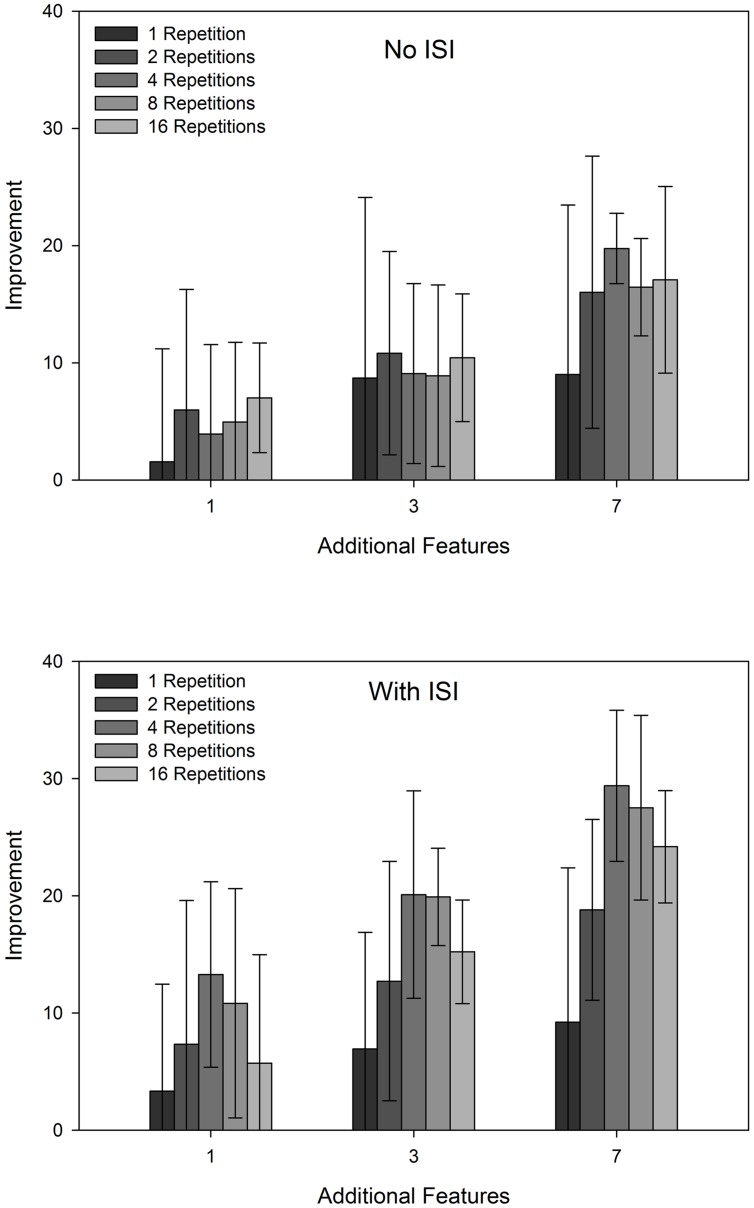
**Increase in change detection accuracy associated with additional changing features over the 10 days of each condition as a function of number of additional changes, number of repetitions, and ISI presence.** Increases are calculated as accuracy on trials featuring multiple changed minus accuracy on trials featuring a single change. Error bars depict 95% confidence intervals.

In order to assess the incremental accuracy associated with presentation of additional changing line features, the increase in accuracy was computed by subtracting accuracy on the baseline (one-feature change) condition from each of the subsequent conditions (2-, 4-, and 8-feature changes). Then a 3 (additional changes: 1, 3, 7) × 5 (repetitions: 1, 2, 4, 8, 16) × 2 (ISI: present, absent) repeated measures ANOVA was run on the calculated increases. All three main effects were significant, and the influence of each variable can be seen in **Figure 3**. The main effect of additional changes indicated that additional changes on each trial produced progressively greater increases in accuracy, *F*(2,10) = 49.568, *p* < 0.001, $\eta_{p}^{2}$ = 0.908. The main effect of repetition indicated that there was greater improvement on longer trials, *F*(4,20) = 3.506, *p* = 0.025, $\eta_{p}^{2}$ = 0.412. Finally, the main effect of ISI indicated that trials with an ISI showed more improvement than trials with no ISI, *F*(1,5) = 10.957, *p* = 0.021, $\eta_{p}^{2}$ = 0.687.

The 2-way interaction between repetitions and ISI was significant, indicating that additional repetitions benefitted trials with an ISI more than trials without one, *F*(4,20) = 2.936, *p* = 0.046, $\eta_{p}^{2}$ = 0.370. The other 2-way interactions and the 3-way interaction were not significant: additional changes × repetition, *F*(8,40) = 1.601, *p* = 0.155, $\eta_{p}^{2}$ = 0.243; additional changes × ISI, *F*(2,10) = 1.748, *p* = 0.223, $\eta_{p}^{2}$ = 0.259; additional changes × repetition × ISI, *F*(8,40) = 0.904, *p* = 0.522, $\eta_{p}^{2}$ = 0.153.

**FIGURE 4:**
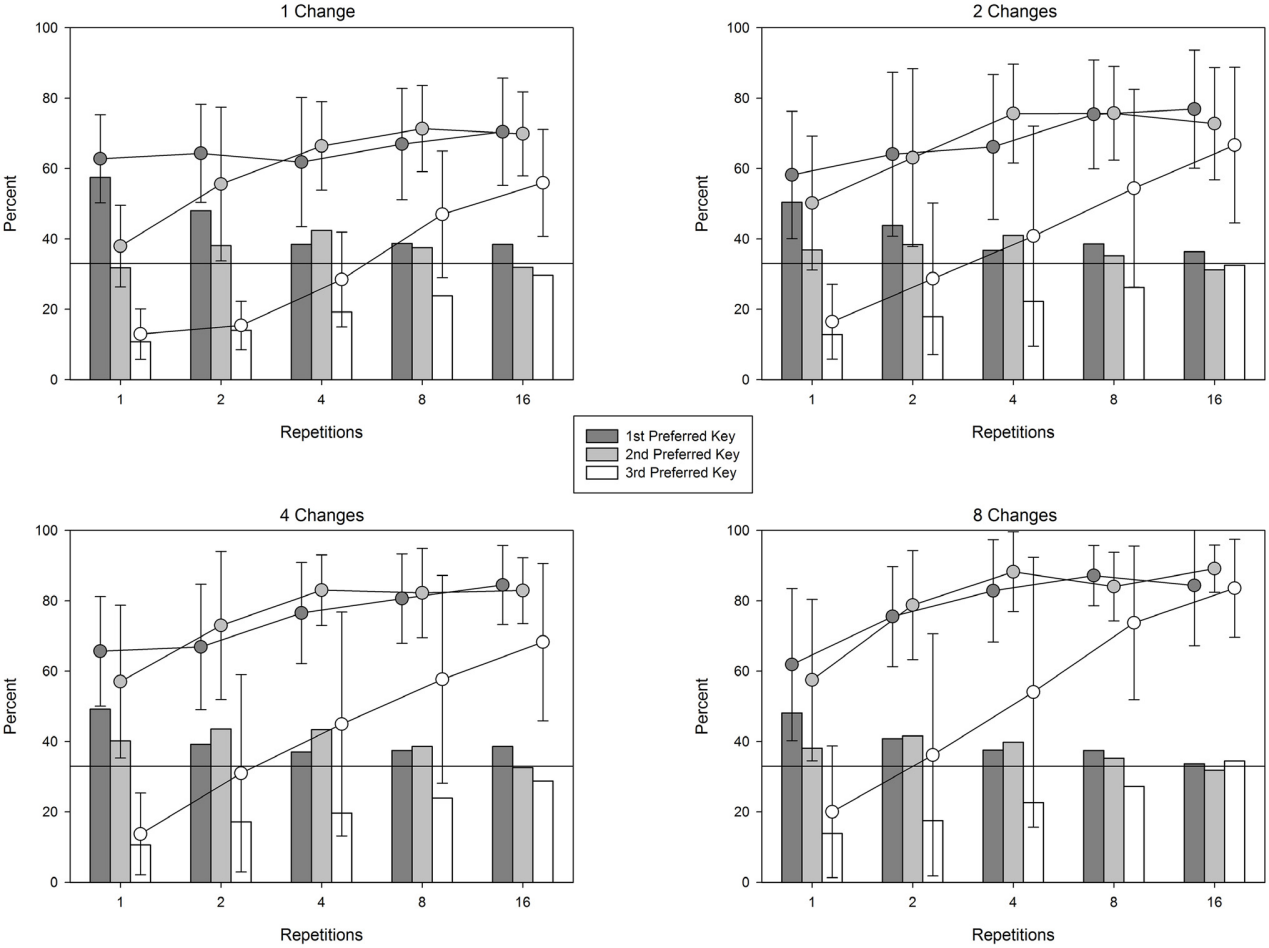
**Analysis of position bias across different numbers of repetitions and the resulting changes in performance during each condition.** Vertical bars represent the percentage of choices on the first, second, and third preferred keys. Key preferences were different for each bird and were determined by the total number of choices on each key for each individual bird. Lines represent the percentage of correctly detected changes presented on each key. Error bars depict 95% confidence intervals.

A secondary pattern not reflected in the above analyses is that each of the birds developed a position bias, distributing their responses unevenly across the three response keys. Vertical bars in **Figure 4** show this position bias as key preferences on trials of different lengths (numbers of repetitions) and during each of the four conditions. Key preferences were determined separately for each bird based on the overall proportions of responses during the entire experiment. As can be seen in the figure, the position bias was quite strong on shorter trials, and gradually weakened as trials became longer. This is confirmed by a 3 (key: first, second, and third preferred) × 5 (repetitions: 1, 2, 4, 8, 16) × 4 (changes: 1, 2, 4, 8) × 10 (session: 1–10) ANOVA. The main effect of key preference was significant, *F*(2,10) = 14.299, *p* = 0.001, $\eta_{p}^{2}$ = 0.741. The interaction between key preference and repetition was also significant, *F*(8,40) = 13.345, *p* < 0.001, $\eta_{p}^{2}$ = 0.727. The main effect of changes, and all interactions involving changes were not significant, *F* < 1.073, *p* > 0.411. The main effect of session, and all interactions involving session were not significant, *F* < 1.102, *p* > 0.304. Thus, it appears that pigeons adjusted their distributions of responses as time elapsed during a trial, but those key preferences were not influenced by number of changes, and the pattern of key preferences did not change significantly across days within a condition.

In order to assess the influence of this dissipating position bias on accuracy, accuracy was computed separately for each response key based on the same individually identified key preferences (shown as lines in **Figure 4**). Note that while chance accuracy is 33% overall, the bars depicted in the figure make for a more sophisticated indicator of chance that takes into account the uneven distribution of responses across keys. A bird responding randomly within the constraints of its position bias should produce correct responses on a key (points on lines) that approximate its overall allocation of responses to that key (bars). For example, in the 1-change condition (top left panel) birds were correct on approximately 60% of single-repetition trials that presented changes on their preferred key, a figure that is reliably better than chance performance of 33% (see the depicted 95% confidence interval relative to the 33% reference line). However, they were able to do so only because they allocated a similarly high percentage of responses on that preferred key. This is in contrast to trials with more repetitions, where birds maintained comparable levels of accuracy on the preferred key, but did so while allocating fewer pecks to that key. Note that because the 95% confidence intervals (error bars) for two or more repetitions do not overlap with the bars depicting response bias, accuracy level is unlikely to be solely based on that response bias.

The pattern of data depicted in **Figure 4** shows how key preference, change salience, and repetition all contribute to change detection. Pertaining to key preference, note that accuracy to detect changes on the first- and second-preferred keys are relatively close throughout. Change detection accuracy on the least-preferred key lags behind the first two, but catches up by the sixteenth repetition. Simultaneously, change salience has a powerful effect: as the number of changing features (change salience) increases, the ceiling for each key (accuracy achieved at the maximum number of repetitions) rises. Finally, accuracy generally rises along with the number of repetitions, but the increase is modulated by the other factors. When four or more changes are presented, accuracy on the first-and second-preferred keys is better than chance by the second repetition, whereas the least-preferred key requires at least eight repetitions. When two or fewer changes are presented, additional repetitions (four or more) are required for the first- and second-preferred keys to exceed chance. Thus, it appears that change detection is a process that occurs over time. As time elapses over the presentation of additional repetitions, pigeons are able to more reliably identify changes (accuracy increases), and can do so across a wider range of locations (three keys rather than two).

## Discussion

This experiment investigated the importance of change salience and timing on pigeons’ ability to detect changes. Results indicated that larger changes were easier to detect, and this basic pattern is the same one that has been previously demonstrated in human participants: if the change in a flicker task involves a greater number of features, it is more likely to be noticed by human participants (Smilek et al., 2000). Our results lead us to the same conclusion: pigeons were better able to identify changes involving additional features. Furthermore, change detection became more effective over time. As additional repetitions were presented, overall accuracy increased, and pigeons’ ability to detect changes expanded to encompass more locations.

The experiment reported here is an extension of the first demonstration of change blindness in pigeons (Herbranson et al., 2014). Though these are the same birds as in that initial research, several important variables were manipulated without changing the primary finding (better on accuracy without an ISI), supporting the idea that the phenomenon of change blindness is robust, and not dependent on a precise set of conditions. Furthermore, it refines those methods, and provides a factor (change salience) than can be used to increase low levels of accuracy on what is a fairly complex task.

Change salience in this experiment was manipulated as the number of changing lines on a particular trial. Note, however, that the number of changing lines might covary with other stimulus characteristics. Consider that on some trials, birds might have been able to detect change based on overall differences in brightness between the original and modified displays if they consisted of different numbers of lines (See bottom panel of **Figure 1**). While this is indeed a possibility, it is tenuous as an overall explanation of pigeons’ performance for several reasons. First, the lines were quite thin, and contributed little to the overall brightness of the operant chamber, especially in the context of a comparably bright houselight. Second, such a strategy would be completely useless on a large percentage of trials – specifically those on which the original and modified displays consisted of the same numbers of lines at different orientations (i.e., when the generation of the alternate display involved adding some line features from the original display while subtracting others; see top panel of **Figure 1**). Third, performance on ISI trials was greater than chance, even though the two different stimulus displays were separated by a blank ISI. The ISI is critical since its brightness would by definition differ from each display by more than the displays could possibly differ from each other. Thus, large changes in brightness would be present and detectable on literally every key during every ISI trial. Finally and most importantly, even if pigeons used a strategy that was based either partly or entirely on stimulus brightness, the major conclusions pertaining to change detection still hold. That is, this remains a *change detection* task, whether birds are detecting changes in the presence of line features or changes in brightness. Additional changing line features makes the difference between the two displays more salient, whether that salience is due to additional visible features or due to a greater difference in illumination (or some other factor).

The previous point underscores some aspects of this experiment that remain uncertain. First, our results do not reveal exactly what aspect of the stimulus pigeons were using to identify changes. Each line feature was different from others in both orientation (the angle of the line) and location (the space occupied by the line), and birds could have used either (or both) to identify changes. It is also possible that some stimuli featuring multiple changes could have produced apparent motion (consider for example, a modified stimulus created by adding one line feature to the original, and deleting an adjacent one). Future research using different kinds of stimuli may help to disentangle these various possibilities. Second, it is not certain what cognitive processes were utilized by pigeons. Pigeons could, for example be using visual short-term memory (or visual working memory) to compare stimuli across the ISI. Similar kinds of change detection tasks have been used quite effectively to study visual short term memory in pigeons and monkeys (Cook et al., 2003; Elmore et al., 2012; Leising et al., 2013), though with different stimuli and time intervals. However, given the short ISI duration in the present experiment it is possible that pigeons instead used sensory memory to compare successive images. The difference might have some important implications, pertaining to the role of attention in change detection, and the nature of representation of objects and scenes (see Rensink, 2002).

Despite those uncertainties, these data might provide some initial insight into the cognitive processes that are at work as pigeons detect changes to their visual environment. Tovey and Herdman (2014) proposed a 3-stage model for human change detection (with pre-processing, feature-extraction, and identification stages operating in sequence). They concluded that large changes could be identified at the second, feature-extraction stage, whereas small changes were identified later, in the identification stage. Indeed, our data are also consistent with such a model. Small changes (those consisting of one feature) produced a gradual and consistent increment in accuracy with repetition, extending to even the longest (16 repetition) trials (see **Figure 2**). This is what one would expect from the identification stage, which requires focused attention, and would presumably operate until either a specific changing feature is identified, or a trial ends. Repetitions of larger changes, on the other hand (four or eight features) produce little improvement in accuracy beyond four repetitions (see **Figures 2** and **3**). Note that a value of four repetitions coincides with the minimum number tested here that would allow for even brief individual consideration of each of the three display keys. The likelihood that the feature extraction stage is sufficient to identify the correct key would be naturally dependent on repetitions and the number of changes (supported by the data), but should not be assisted by additional repetitions once each location has been processed. Thus, it would seem possible that a similar stage-based model might apply to pigeon change detection. Further research on the interactions between easily manipulable factors that presumably operate at early stages (such as stimulus quality or discriminability) and at later stages (such as familiarity or learned associations) would be an ideal test of the model’s applicability to pigeons.

Since change by definition occurs over time, this change detection task tells us some important things about temporal cognition in pigeons. First, the timing of a change has a powerful influence over detection. Instantaneous changes are easier to detect than those obscured by a temporally coordinated ISI. Furthermore, pigeons (like humans) appear to engage in a serial search process, allowing them to detect changes in a larger region of space as time passes. Note however, that additional time does not constitute an absolute advantage. ISI trials are longer in duration than no-ISI trials involving the same number of repetitions, yet they produce lower levels of change detection accuracy. Thus, there appear to be two aspects of time that contribute to successful change detection. Additional repetitions (occurring over time) enhance change detection, whereas interruption of continuity by an ISI (also occurring over time) inhibits it. It is not yet entirely clear how these two factors interact. Herbranson et al. (2014) showed that shorter ISI durations had a progressively weaker effect on accuracy. If pigeons do engage in a serial search process, then increasing the rate of presentation might weaken the repetition effect by limiting the number of locations that can be considered per repetition. Hagmann and Cook (2013) studied a related temporal aspect of change detection, using a more precise manipulation of change. Unlike the discrete changes in the present experiment, they presented pigeons with stimuli that changed continuously in brightness, at various rates. Birds were able to discriminate changing stimuli from constant ones, but accuracy was significantly influenced by rate of change. Future research should delve further into the temporal aspects of change detection to create a more complete understanding of the cognitive process at work.

Given the results indicating that effective search area expanded with additional repetitions, this procedure might also be an effective one for investigating sequential search strategies. While there was no indication that pigeons’ strategies changed across days, individual birds did have different stable key preferences, indicating that they went about the search process in different ways (i.e., beginning on different keys and then progressing to others in different orders). Presumably those individual key preferences arose during pretraining and persisted through the conditions and sessions reported here. Note that because changes were equally likely to appear on any of the three keys, such variations in search strategies should have no effect on accuracy over the long run. However, if birds are indeed performing a systematic search, then probabilistically cueing upcoming change locations (either through base-rate manipulations or trial-by-trial priming) might bias birds toward a specific strategy that would increase accuracy by allowing them to begin their search at the most likely change location. This could be another possible means of increasing accuracy on the task, and perhaps expanding it to study other cognitive processes.

## Conclusion

Change detection is a fundamental aspect of temporal cognition that can and has been investigated in both humans and pigeons. So far, the factors that influence change detection and failures of change detection (i.e., change blindness) appear to be similar in both species. These factors include the timing and salience of a change. Furthermore, the influence of these factors change over the course of a trial, indicating that change detection may provide some important insight into the temporal aspects of cognition.

## Conflict of Interest Statement

The author declares that the research was conducted in the absence of any commercial or financial relationships that could be construed as a potential conflict of interest.
